# Active children through individual vouchers – evaluation (ACTIVE): protocol for a mixed method randomised control trial to increase physical activity levels in teenagers

**DOI:** 10.1186/s12889-017-4554-7

**Published:** 2017-07-11

**Authors:** Michaela James, Danielle Christian, Samantha Scott, Charlotte Todd, Gareth Stratton, Sarah McCoubrey, Julian Halcox, Suzanne Audrey, Elizabeth Ellins, Sinead Brophy

**Affiliations:** 1 College of Medicine, Swansea University, Data Science Building, Swansea, SA2 8PP UK; 2 Department of Sport and Physical Activity, Edge Hill University, St Helens Road, Ormskirk, Lancs L39 4QP UK; 3 College of Engineering, Bay Campus Swansea University, Fabian Way, Crymlyn Burrows, Skewen, Swansea, SA1 8EN UK; 4 City and County of Swansea Council, Room 153, Guildhall, Swansea, SA1 4PE UK; 5 College of Medicine, Swansea University, Singleton Park, Swansea, SA2 8PP UK; 6 School of Social and Community Medicine, Bristol, BS8 2PS UK

**Keywords:** Fitness, Physical activity, Peer mentor, Teenagers, Voucher, Mixed methods

## Abstract

**Background:**

Many teenagers are insufficiently active despite the health benefits of physical activity (PA). There is strong evidence to show that inactivity and low fitness levels increase the risk of non-communicable diseases such as coronary heart disease (CHD), type 2 diabetes and breast and colon cancers (Lee et al. Lancet 380:219–29, 2012). A major barrier facing adolescents is accessibility (e.g. cost and lack of local facilities). The ACTIVE project aims to tackle this barrier through a multi-faceted intervention, giving teenagers vouchers to spend on activities of their choice and empowering young people to improve their fitness and PA levels.

**Design:**

ACTIVE is a mixed methods randomised control trial in 7 secondary schools in Swansea, South Wales. Quantitative and qualitative measures including PA (cooper run test (CRT), accelerometery over 7 days), cardiovascular (CV) measures (blood pressure, pulse wave analysis) and focus groups will be undertaken at 4 separate time points (baseline, 6 months,12 months and follow-up at 18 months). Intervention schools will receive a multi-component intervention involving 12 months of £20 vouchers to spend on physical activities of their choice, a peer mentor scheme and opportunities to attend advocacy meetings. Control schools are encouraged to continue usual practice. The primary aim is to examine the effect of the intervention in improving cardiovascular fitness.

**Discussion:**

This paper describes the protocol for the ACTIVE randomised control trial, which aims to increase fitness, physical activity and socialisation of teenagers in Swansea, UK via a voucher scheme combined with peer mentoring. Results can contribute to the evidence base on teenage physical activity and, if effective, the intervention has the potential to inform future physical activity interventions and policy.

**Trial registration:**

ISRCTN75594310 (Assigned 06/03/2017).

## Background

Being active in adolescence is associated with many health benefits [1–6] and physical activity (PA) levels established during this time are likely to be taken into adulthood [2]. However, reports show that many adolescents are not sufficiently active to achieve these benefits [2, 7]. Government recommendations for PA suggest adolescents should be engaging in at least 60 min of moderate to vigorous physical activity (MVPA) every day [8]. A large proportion of young people do not meet this recommendation in Wales [9], with recent evidence showing that only 11% of girls and 20% of boys are sufficiently active [10]. This is concerning, as there is strong evidence to show the increased risk of non-communicable disease such as CHD, reduced well-being and shortened life expectancy resulting from inactivity and low aerobic fitness levels [11, 12].

Given that 7 million people in the United Kingdom are fighting CV diseases, [13] with physical inactivity a major risk factor, the development of effective interventions to promote activity in adolescence is of urgent public health concern [14]. It is reported that one of the main barriers to PA for teenagers is accessibility (e.g. cost and lack of local facilities) [8, 15], particularly for teenagers from disadvantaged backgrounds [4]. There is also a population trend towards spending more time inside, where technology can increase screen time and sedentary behaviours [3, 6].

The Active Children through Individual Vouchers – Evaluation Project (ACTIVE), funded by the British Heart Foundation (BHF) [16], aims to tackle these barriers and increase PA by giving teenagers vouchers to spend on activities of their choice. The project encourages teenagers to access existing provisions or generate their own in order to tackle accessibility issues and create the opportunity to participate in desired activities [15]. Evidence has shown that empowering teenagers to make their own choices over which activity they engage in, the location where they engage, and the people they participate with, can improve activity levels [17]. ACTIVE will also encourage teenagers to express the importance of choice and empowerment in advocacy meetings with stakeholders. As a result, the project aims to enhance socialisation and peer support, in order to facilitate PA uptake. This has been positively associated with teenage activity levels [18].

A voucher based intervention to increase PA in the UK has been previously tested amongst adults [19, 20], however, it remains uncertain whether a similar approach could work with teenagers. Financial incentives have been previously tested within a variety of populations [21–24] and have been effective in increasing physical activity levels. However, these focus on financial rewards rather than activity enabling vouchers. The ACTIVE Project aims to investigate whether a multi-component voucher based scheme can positively influence teenagers to become more physically active and improve their cardiovascular fitness.

## Feasibility study

The ACTIVE feasibility study [15] was a mixed method cohort and process evaluation study of one school in a deprived area of Swansea, South Wales. The school was classed as deprived based on: i) the number of pupils eligible for free school meals (FSM) (54% at time of study) [25], ii) the area’s eligibility for community-based initiatives and funding (e.g. Communities First) [15] and iii) the location in one of Wales’ most deprived areas for children [26]. The study measured outcomes at three different time points (baseline, 5 months (during intervention) and 12 months (follow-up)). All Year 9 pupils in the school were given activity vouchers (*n =* 115; 13.3 ± 0.48 years; 51% boys).

The project found increases in MVPA and decreases in sedentary behaviour, suggesting a positive impact from voucher usage [15]. Process evaluation based on the RE-AIM Framework [27] demonstrated that ACTIVE was well received by pupils and teachers and was a feasible approach to increasing PA amongst adolescents from low socio-economic backgrounds.

Adjustments were made to the ACTIVE protocol following outcomes, process evaluation and recommendations from funding partners. These adjustments were made to further improve the project and increase its sustainability, prior to conducting a Randomised Controlled Trial (RCT) in order to assess effectiveness rather than its feasibility.

## Aims

The current study builds upon the feasibility study by examining the effect of a multi-component voucher based intervention on the cardiovascular fitness and MVPA levels of teenagers aged 13–14 years in seven schools in Swansea (4 intervention and 3 control schools). The specific aims of the ACTIVE Project are as follows:

### Primary aims

1. To examine evidence of the effect of a multi-component intervention in improving cardiovascular fitness based on Cooper Run Test score.

### Secondary aims

2. To examine evidence of the effect of a multi-component intervention in reducing time spent sedentary, as measured by 7-day accelerometry.
3. To determine the effectiveness of the ACTIVE intervention to improve the following secondary outcomes:

- The amount of teenagers meeting the recommendation of 60 min of MVPA per day.
- Cardiovascular health (blood pressure and pulse wave analysis (PWA) an indicator of arterial stiffness).
- Exercise motivation (BREQ-2).
- The characteristics of teenagers who engage with the scheme, particularly among high risk groups to determine what works for whom, why and in what contexts.

4. To examine evidence of the effect on sustained local investment in implementing recommendations of teenagers in promoting PA and cost-effectiveness.
5. To provide evidence of whether ACTIVE can have a sustainable effect on fitness and PA (18-month follow-up)
6. To provide evidence that ACTIVE can be implemented by the local council with future rollout to other areas.
7. To undertake data linkage of quantitative measures through the Secure Anonymised Information Linkage (SAIL) databank [28] to analyse the effects of physical activity levels on educational attainment and GP visits.

## Design

ACTIVE is a mixed methods randomised controlled trial based in state secondary schools in Swansea, South Wales. Schools will be approached to take part due to their: i) location in one of Wales’ most deprived areas and ii) location in a Communities First catchment area [29, 30]. Randomisation will occur prior to baseline data collection with schools randomised into either the intervention arm or control arm. Due to the nature of the study, participants will be aware of which arm they have been allocated to.

The College of Human and Health Science Ethics Committee at the College of Medicine, Swansea University granted ACTIVE ethical approval on 12/05/2016.

## Participant recruitment

Following initial school recruitment, participants will be recruited for primary and secondary outcome measures via Year 9 assemblies. During the assemblies, researchers on the project (DC & MJ) will provide information about the project and answer any questions before distributing project information, parental consent and pupil assent forms after the assembly. Once consent and assent has been obtained, participants will be eligible to partake in the study.

## Intervention

The ACTIVE intervention consists of three different components; a) physical activity vouchers, b) peer mentor scheme, advocacy meetings and pupil-led video production, and c) support worker engagement.

### Physical activity vouchers

The intervention involves provision of vouchers equating to a monetary value of £20 (four vouchers in increments of £5) per pupil each month for 12 months. They are to be distributed in schools with the purpose of being used to; i) spend on existing activities, ii) fund new activities in the schools or communities such as fitness classes, and iii) purchase sporting equipment for themselves or a club. The vouchers are to be treated similar to a cash transaction but without the delivery of change, in order to prevent the purchase of non-PA based items. At the end of each month, vouchers will be collected from each provider with an accompanying invoice for reimbursement of fees. This transaction process has been informed by the ACTIVE feasibility study [7]. Activity providers (for example, leisure centres and sports clubs) have been recruited during the development stages of the project and will continue to be recruited throughout the intervention based on pupil suggestions.

### Peer mentoring scheme, advocacy meetings and pupil-led video production

Prior to baseline measures, pupils from each school will be asked to identify key influencers to be peer mentors (10 from each school). A peer nomination questionnaire was given to all pupils in the year and those who received the most nominations were invited to be peer supporters [31]. These individuals will receive training to be ‘peer supporters’ from the Active Young People team at Swansea Council and a student from the PR and Marketing Taught Masters course at Swansea University. The role of peer supporter is to encourage the uptake and sustainability of physical activity. This approach has been underpinned by ASSIST (A Stop Smoking in Schools Trial); an intervention which involved training Year 8 students to promote the benefits of not smoking amongst their peers. [32].

Peer mentors will be encouraged to produce videos throughout the intervention to explain why activity is important to them, the barriers to being active and any recommendations to improve activity for teenagers. These will help provide an innovative way to display the work of the ACTIVE intervention at advocacy meetings. These videos will be uploaded to YouTube and shown to local stakeholders and providers. Using an ACTIVE profile, videos will be recorded via ‘Snapchat’, a popular image-messaging app. Studies have shown that ‘Snapchat’ is used to facilitate relationships and ‘bonding’ amongst social circles [33, 34] and combined with its use of filters and text will create a novel way of displaying physical activity perceptions of this age group.

Throughout the ACTIVE intervention, advocacy meetings will be held with key stakeholders in order to promote sustainable investment for the provision of physical activities in the local community. These meetings will occur at 6 months and 12 months and involve discussion of needs, barriers to activity and changes that could improve teenager’s access and uptake from the peer mentors’ point of view.

### Support worker engagement

A support worker will regularly attend the participating schools to increase pupil awareness of what is available in the area, provide advice on how to access activities and encourage pupils to design new activities or attract new coaches to the area. The support worker will audit the activities available and monitor voucher usage monthly to identify those engaging well and those not engaging with the project. Each month, the support worker will host drop-in style sessions in school lunchtimes so that pupils can liaise regarding their ideas and current engagement with the vouchers, and raise any questions or clarify any issues they may have. Feedback from these sessions will be used to target new activity providers that pupils have specifically expressed interest in.

Regular contact with the schools will aid communication, and maintain a presence to help pupils and teachers feel supported by the project. Assemblies will be held at the schools, to directly update the year group with important information regarding their vouchers and activity providers, also providing a general summary. The support worker will also communicate with local activity providers to create new activity options and ensure vouchers remain redeemable throughout the 12-month intervention.

### Control schools

Control schools are encouraged to continue with their usual practice throughout the duration of the study. They will receive a mindfulness course for staff or pupils because of their participation in the study at baseline, 6 months, 12 months and 18 months.

## Data collection

**Fig. 1:**
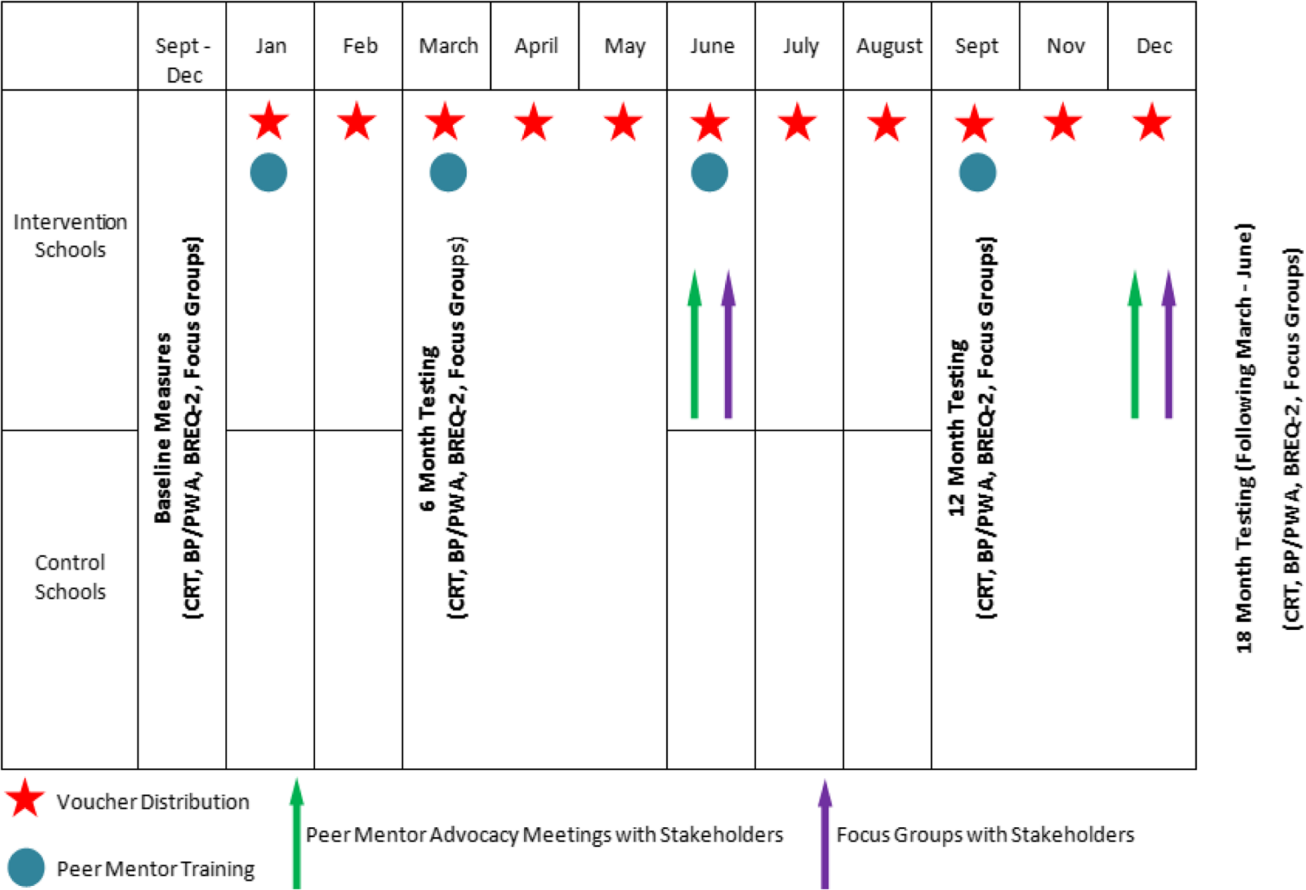
Data collection and Intervention time scale

Data collection periods will take place at three time points: baseline, 6 months, 12 months and 18 months follow-up for both intervention and control schools (Fig. 1). Quantitative measures of fitness, PA, motivation and cardiovascular health will be assessed at all four time points using the measures described below, in addition to focus groups and interviews to assess qualitative aspects of the project.

### Cardiovascular fitness (Cooper run test)

The CRT is a 12-min walk/run test that will be performed at all four time points to assess cardiovascular fitness. The validity of the CRT as a predictor of aerobic fitness has been tested by numerous studies in both young males and females [35–37]. The test will be performed as part of normal PE lessons in the schools to avoid disruption to school timetables.

### Physical activity (Actigraph accelerometers)

Physical activity will be measured via Actigraph GT3X+ accelerometers (Actigraph, Pensacola, Florida, United States), a tri-axial accelerometer which has previously been used in adolescents both for hip and wrist placement [38, 39]. The accelerometers will be worn on the non-dominant wrist of the participant, as opposed to the hip, for improved compliance [40]; a methodology adopted by the recent National Health and Nutrition Examination Survey (NHANES) between 2011 and 2012. The Actigraphs will be worn for seven full days at all times, apart from bathing or swimming, and will be set to record at a frequency of 30 Hz.

### Motivation (exercise motivation questionnaire (BREQ-2))

The modified Behavioural Regulation in Exercise Questionnaire (BREQ-2) is a 19- item questionnaire that provides scores for amotivation and external, introjected, identified and intrinsic regulation. It is an accessible questionnaire, clearly written to aid pupils understanding of the questions. The BREQ-2 has been noted to obtain good psychometric information [41] and has been previously used to provide information regarding teenager’s motivation to exercise [42–44]. The questionnaire will be administered at all four time points.

### Cardiovascular health (blood pressure and pulse wave analysis)

Blood pressure will be measured through a standardised upper arm cuff methodology using a sphygmomanometer (Omron M2 monitor, OMRON Healthcare UK Ltd., United Kingdom). Participants will be seated for a minimum of five minutes to allow them to be sufficiently rested prior to any measures being taken. The Omron cuff will be positioned on the upper left arm, with the midline of the cuff positioned over the brachial artery, and the arm out straightened, resting gently on a table so as not to influence the blood pressure reading [45]. The cuff size will be chosen in accordance with recommendations to ensure adequate fit for all participants [45]. The cuff will be inflated until a blood pressure recording appears on the Omron M2 monitor screen, at which point the cuff will deflate. This process will be repeated twice more, allowing the average of the three measures to be taken. Should any of the measures be very different (+/− 5 mmHg) an additional measure will be taken.

To further assess vascular function, non-invasive measurement of pulse wave analysis will be undertaken as an indicator of arterial stiffness [46]. Pulse wave analysis will be assessed using the Vicorder (Skidmore Medical Limited, Bristol, United Kingdom). Participants will be seated and a SC10 Hokanson cuff positioned around their upper left arm. Once the participant has rested for five minutes, the cuff will be gradually inflated according to an inbuilt automated protocol, during which the brachial artery pulse-pressure waveform is recorded. Central augmentation pressure and augmentation index are determined from the waveform using a transfer function integral to the software. This process will be performed a second time, if both measures of augmentation pressure are within ±5 mmHg of each other and augmentation index are within ±5% the two measures are accepted, if not a third reading is taken and a mean of all 3 taken.

### Adolescents’ views (focus groups)

Semi-structured focus groups will consist of 6–8 pupils with boys and girls in separate groups (two focus groups per school). Participants will be asked their opinions regarding what physical activity is, its barriers and what could be done to improve activity in their areas. Intervention schools will discuss the ACTIVE project specifically (See Topic Guides: Appendix one). Baseline focus groups will consist of pupils of randomly selected consented pupils. After this, participants purposively selected from consented pupils to gain a variety of viewpoints from those engaging well with the intervention and those who are not. The focus groups will provide ACTIVE with a greater understanding of the mediating factors that influence teenage physical activity. These will also help provide context to the quantitative measures from baseline to 18 months.

In addition to focus groups with teenagers, focus groups and interviews will be held with stakeholders (e.g. Active Young People Officers from the local council and teachers from intervention schools) to inform the process evaluation of ACTIVE. These will be held at the 6 month, 12 month and 18 months data collection time points.

## Analysis

CONSORT guidelines will inform the analysis and presentation of the study [47]. Multilevel regression analyses will test the effect of the intervention on our primary and secondary outcome measures in comparison with the control arm. We will adjust for baseline, 6 month, 12 month and 18 month follow-up scores and baseline characteristics (e.g. sex). Focus groups will be analysed via thematic analysis in order to identify key themes from discussions with pupils involved in the project [48]. Quantitative measures will be linked to the SAIL databank to analyse the impact of physical activity levels on educational attainment and GP visits. COREQ (Consolidated Criteria for Reporting Qualitative Research) will be followed for the qualitative aspects of the research.

This RCT will examine change in the intervention group compared to controls. Findings from the feasibility study showed that sedentary behaviour reduced by 65 min (95% CI: 12.0 to 117.6) from baseline (*n* = 75). A previous study [49] had an intracluster correlation of fitness scores across 10 schools of 0.16. Therefore, estimating a reduction in sedentary behaviour of 65 min (intervention) and 15 min (control) with a standard deviation of 30 min, and an average cluster size of 150 children per school ICC of 0.16, coefficient of variation of cluster sizes of 0.9, power of 80% and significance of 5% would require 450 children per arm in 3 schools in each arm (6–8 schools in total depending on consent rates).

Fitness improved in the feasibility study in the intervention group by 98 m (95% CI: 19 to 177, children ran 1730 m in 12 min at baseline and 1823.3 m at post intervention). Therefore, estimating improvement in cooper run of 98 m (intervention) and 22 m (control) with average cluster size of 150 children per school and ICC of 0.16 (as above) would require 300 children or 2 schools per cluster (3 schools if consent rate is assumed to be 60%).

## Conclusion

ACTIVE is a novel intervention aimed at examining the effect of a multi-component intervention in improving the cardiovascular fitness, arterial physiology and general health, whilst reducing time spent sedentary, in adolescents. Providing insight into the results of the trial, alongside process evaluation can strongly add to the evidence base in this field and can inform future intervention and policy involving teenagers and physical activity.

## Appendix 1

**INTERVENTION FOCUS GROUP TOPIC GUIDE – PARTICIPANTS – PRE-INTERVENTION.**

*Activity 1 (5–10 min):* What is physical activity? What does it mean to you?

*(Ask pupils to discuss/write down what the term means to them).*

How active should people your age be? (60 min recommended per day).

*Question 1:* How active do you think people in your year are? Do you think you are as active as you can be?

*Activity 2 (5–10 min):* What do you see as the current barriers to physical activity? How do you feel about your current levels?

*(Give post-it notes and ask pupils to list 5–6 barriers to activity. Then rank these barriers in order of most common barriers and discuss the reasoning for this order).*

*Question 2:* Why do you think people your age like/don’t like being active? Is there much to do in your area? If so, how accessible are these activities to your age group?

*Question 3:* Vouchers are currently accepted by *(refer to list of participants).* What other activities or providers would you like to see included before we start?

*Question 4:* Having heard the way the scheme is set up; do you think there will be any problems? What do you think we could do about these problems? Do you think we should do things differently?

*Question 5:* What is the best way of letting everyone in the school know about this scheme? When are the best times for the support worker to be available in the school?

*Question 6:* Do you have anything else to add about the project?

**CONTROL FOCUS GROUP TOPIC GUIDE – PARTICIPANTS – PRE-INTERVENTION.**

*Activity 1 (5–10 min):* What is physical activity? What does it mean to you?

*(Flipchart/post-its – ask pupils to discuss/write down what the term means to them).*

How active should people your age be? (60 min recommended per day).

*Question 1:* How active do you think people in your year are? Do you think you are as active as you can be?

*Activity 2 (5–10 min):* What do you see as the current barriers to physical activity? How do you feel about your current levels?

*(Give post-it notes and ask pupils to list 5–6 barriers to activity. Then rank these barriers in order of most common barriers and discuss the reasoning for this order).*

*Question 2:* Why do you think people your age like/don’t like being active?

*Question 3:* Is there much to do in your area? If so, how accessible are these activities to your age group?

*Question 4:* Are your friends/family active? Does this influence you?

*Question 5:* What is the best way to get people your age active?

*Question 6:* Do you have anything else to add?
